# Thermo-Hydro-Glycol Ageing of Polyamide 6,6: Microstructure-Properties Relationships

**DOI:** 10.3390/polym14194097

**Published:** 2022-09-30

**Authors:** Clément Laügt, Jean-Luc Bouvard, Gilles Robert, Noëlle Billon

**Affiliations:** 1 MINES Paris, PSL Reaserch University, Center for Materials Forming (CEMEF), CNRS UMR 7635, CS 10207, 06904 Sophia Antipolis, France; 2 DOMO Chemicals, POLYTECHNYL France SAS, Avenue Albert Ramboz, 69190 Saint-Fons, France

**Keywords:** PA66, microstructure, mechanical behavior, thermo-hydro-glycol ageing

## Abstract

The microstructural evolutions occurring during the thermo-hydro-glycol ageing of an injection molded PA66 were studied. They were correlated to the evolutions of its mechanical properties. The aged samples were immersed in an antifreeze fluid—mainly composed of water and ethylene glycol—at varying times and temperatures. The aim was to combine an as exhaustive as possible microstructural investigation and a rigorous mechanical analysis. Consequently, the microstructure of the aged and unaged PA66 was assessed through the average molar mass, the diameter of the spherulites, the lamellae thickness, the crystallite’s apparent size, a crystal perfection index, and a crystallinity index. Moreover, a core-skin approach was set up. The mechanical consequences of the microstructural changes were investigated by DMA and tensile testing. The local true strain fields were measured with a digital image correlation system. The temperatures and strain rates of the tests were chosen by referring to the time-temperature superposition principle. It is concluded that the water and ethylene glycol intake resulted in an intense plasticization, the loss of the molar mass resulted in the embrittling of the polymer, and finally, it was identified that the changes of the crystalline structure have an influence on the stiffness of PA66.

## 1. Introduction

Polyamide 6,6 (PA66) is a semicrystalline polymer, widely used in the automotive industry, as a matrix for reinforced thermoplastic parts. It is especially used in the cooling systems of engines. There, it faces high temperatures in the presence of the antifreeze fluid, which is mainly composed of water and ethylene glycol. Over time, these conditions may operate as physical or chemical aging. They also may impact the crystalline microstructure, and eventually the mechanical properties. Three main phenomena are expected to occur: the diffusion of water and ethylene glycol molecules into the amorphous phase [1], the chemical interactions between these molecules and the polymer [2], and eventually, as suggested [3], some crystal modifications.

The amide functions of polyamides favor H-bounds, especially with species such as water and ethylene glycol. The absorption of water and ethylene glycol by PA66, is a well-documented phenomenon [1,4,5]. The interactions between these molecules and the amide functions were confirmed with the FT-IR method [6,7,8]. In parallel, the kinetics of the diffusion process is often described with a Fick law [2,9,10,11,12]. As far as the mechanical behavior is concerned, the interactions between the amide functions and the water or ethylene glycol molecules have an influence on the apparent mobility of the macromolecular chains. It results in the well-known plasticizing effect, which is characterized by a decrease of the α relaxation temperature $T_{\alpha }$ [2,10,11]. The intensity of this decrease depends on plasticizer content [13,14,15].

In addition to the above-mentioned physical interactions, some chemical interactions between the polymer and the absorbed species can also be observed. PA66 is indeed created by a polycondensation/hydrolysis balanced reaction. The polycondensation involves a diamine and a diacide, and it generates water as a sub-product. The presence of water in the amorphous phase of the polymer may shift the equilibrium of this balanced reaction in favor of the hydrolysis reaction. When it occurs, water molecules react with the amide functions of the polymers, resulting in the scission of the macromolecular chains. Two end groups are indeed created instead of the amide function: an acid and an amine. It leads to a decrease of the average molar mass [2,16,17,18,19]. In that field, Ledieu [2] and Jacques et al. [16] suggested a kinetic model for the hydrolysis of PA66 and PA11, respectively. The main impact of the molar mass decrease is the embrittlement of the polymer [2,18,20] characterized by a decrease of the ultimate strain in tension. The amide functions of the PA66 can also react with the ethylene glycol molecules. This reaction called glycolysis could break the macromolecular chains, creating some ester and amine functions. Some authors identified the occurrence of this reaction through the evolution of the FT-IR peaks corresponding to the ester and amine functions [21,22,23]. Kim et al. [21], and Huckowski et al. [23] also measured the decrease of the PA66 average molar mass within the glycolysis time and suggested a model for this reaction. However, the glycolysis reaction was not observed at temperatures below 200 °C without the use of a catalyst. Such temperatures were not reached in the present study. Hence, the glycolysis reaction was not expected. Finally, when immersed in water with oxygen, polyamide can also undergo a chemical degradation due to oxidation [24]. This reaction results in the random chain scission, and in the crosslinking of the polymer. This latter phenomenon can be observed through the increase of the polydispersity index [24,25]. It should be noted that the present paper neither focuses on which specific chemical reaction takes place nor on their kinetic, but on their microstructural consequences.

As far as the crystal phase is concerned, annealing processes can generally have an impact on the crystal properties of semicrystalline polymers such as crystallinity, apparent size of the crystallites, lamellae thickness and phase organization. These crystal properties may have an influence on the polymer’s mechanical behavior, especially on the stiffness and ductility [26,27,28].

Finally, the injection molding process does not generate a homogeneous microstructure. Depending on its location in the mold, the material does not undergo the same thermo-mechanical history in terms of temperature, cooling rate, pressure, or shear rate. These differences in the thermo-mechanical conditions may have an influence on the microstructure and on the mechanical properties [27,29,30].

The present survey had two main goals. The first, to assess the microstructure evolution of an injection molded PA66 when it undergoes high temperatures in the presence of water and ethylene glycol. The second, to investigate the eventual mechanical consequences of the microstructural changes. In the literature, only a few studies deal with the microstructure evolution of polyamide during thermo-hydro-glycol ageing, and these focused on the average molar masses. The present survey is a novelty as it gives a thorough assessment of the microstructural evolutions involved in the thermo-hydro-glycol ageing of an injection molded PA66. The microstructure was indeed described not only by the average molar mass but also by the crystal properties. Several methods were involved to evaluate the microstructure. The molar mass was measured by size-exclusion chromatography and a crystal index was measured by wide-angle X-ray scattering (WAXS). Moreover, a multiscale approach was set up to describe the crystalline phase. At the spherulite scale, the spherulite diameters were observed and measured by polarized light microscopy. At a lower scale, the lamellae thickness was evaluated by differential scanning calorimetry (DSC) and the crystal apparent size was measured by WAXS. Finally, at the crystal cell scale, a crystal perfection index was estimated by WAXS. A significant part of this survey was also to evaluate the impact of the microstructure evolution on mechanical behavior. It is a unique feature of this study, as it handles at the same time a complete microstructural and a strong mechanical evaluation. Here, the viscoelastic properties were assessed by dynamic mechanical analysis (DMA) measurements. The large strain mechanical behavior was assessed through tensile testing, taking advantage of the time-temperature superposition principle as already suggested in the literature [31,32,33]. The local true strain was measured with a digital image correlation (DIC) system. Finally, to take into account that the injection-molding process does not generate a homogeneous microstructure in the thickness of the samples, a core-skin approach was set up to consider the likely microstructural and mechanical gradient in the thickness of the PA66 samples.

Hence, the present survey is a contribution to the industrial and academic sectors as it gives a thorough assessment of the evolution of PA66 when used in the cooling systems of automotive engines. Moreover, the assessment of the evolution of the crystal properties as well as the molar mass is not common and especially when related to thermo-hydro-glycol ageing. Finally, an in-depth analysis of the microstructural-properties relationships of PA66, dealing at the same time with the plasticization, the influence of the average molar mass, and of the crystal properties, is a novelty, especially when the mechanical behavior is precisely evaluated (using of a DIC system and referring to the time-temperature superposition principle).

## 2. Materials and Methods

### 2.1. Materials

**Figure 1:**
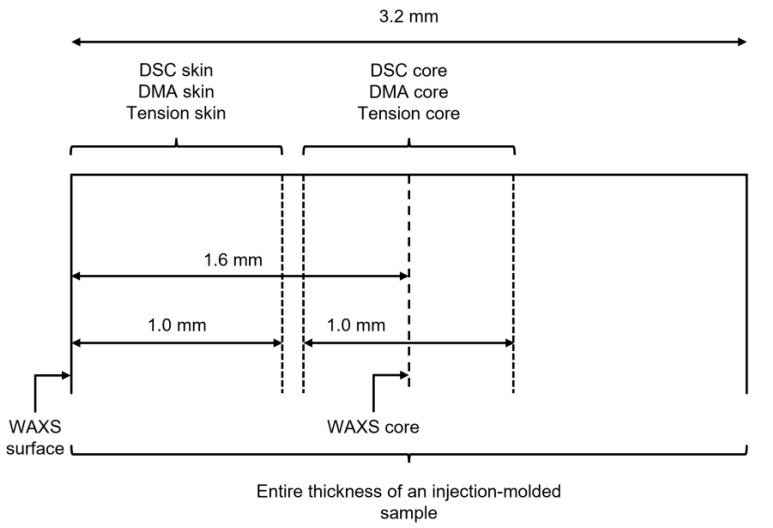
Illustration of the skin-core sampling strategy. The core and the skin samples were tooled from the whole thickness of the plates with a milling machine.

The material was a PA66 Technyl^®^ A218 34NG grade, with a 1.14 density. 3.2 mm-thick injection molded plaques processed from the same batch were provided by the company DOMO Chemicals (Saint-Fons, France). They were injection molded from pellets. The mold temperature was 80 °C. The injection time was 3.92 s, and the injection pressure was 927 bar. The holding time was 10 s, and the holding pressure was 500 bar. The samples were machined in the thickness of the plaques. Reference samples were dried under vacuum at 80 °C for 48 h. Then thermo-hydro-glycol ageing was performed in an autoclave where samples were immersed in an antifreeze bath at different temperatures from 120–140 °C, and during times. At 120 °C and 130 °C samples were aged during 48 h, 168 h, 336 h, 504 h, and 1000 h, and at 135 °C and 140 °C samples were aged during 168 h and 336 h. The antifreeze was a 50%:50% volume mixture of water and ethylene glycol. The glycol was supplied by the company DOMO Chemicals (Saint-Fons, France). In the autoclave, the pressure only depended on the process temperature: no pressure instruction was set. Core/skin measurements were performed by reducing the thickness of the samples with a milling machine: 1 mm thick from the surface in the case of “skin” samples and 1 mm thick in the core for “core” samples. The sampling strategy is illustrated in Figure 1.

### 2.2. Steric Exclusion Chromatography

The average molar masses were determined by the company DOMO Chemicals (Saint-Fons, France) with the size-exclusion chromatography (SEC) method. The samples were taken in the whole thickness of the plaques, including the core and skin areas. Due to confidentiality reasons, the protocol cannot be described in more detail, and the initial molar mass of the unaged PA66 cannot be released. Moreover, only the standardized molar masses are displayed in this paper. They were standardized from the initial molar mass of the unaged PA66.

### 2.3. Polarized Light Microscopy

A Leica (Paris, France) polarized light microscope fitted with a gypsum plate, was used to observe the spherulites from the core to the surface of the molded samples. Thin PA66 slices were cut with a glass-blade microtome. Their thickness was around 100 μm.

### 2.4. Wide Angle X-ray Scattering

**Figure 2:**
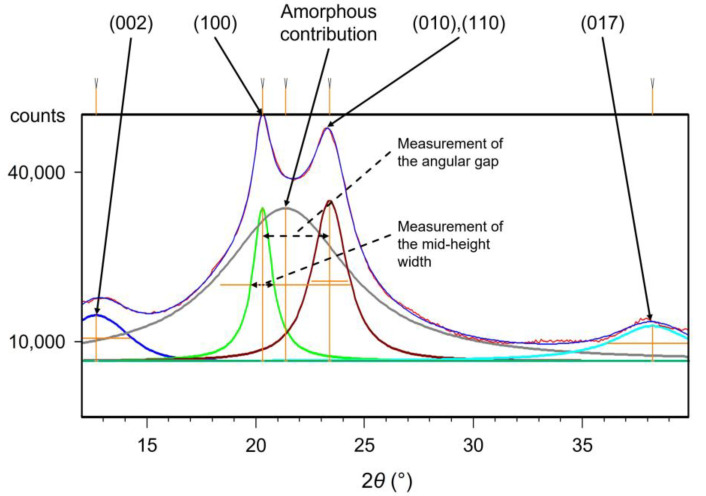
Typical decomposition of a PA66 diffractogram, with the identified plan families.

The WAXS analysis was performed with a Philips (Amsterdam, Netherlands) X’Pert Pro, using CuKα radiation with a wavelength λ = 1.540 Å. A goniometer with a *θ*–*θ* set-up was used. The tests were performed with a 12–40° angular scan and an exposure time of 12 min. After the measurements, the diffractograms were decomposed to identify the amorphous and the crystal phases’ contributions. A flat background was fixed at 80% of the minimum intensity. A typical decomposition is depicted in Figure 2. The peaks can be indexed to their corresponding plan families thanks to the works of Bunn and Garner [34], Starkweather [35,36], and Haberkorn [37]. The peaks were calculated with Pearson VII symmetric functions. The mid-height width of the (100) peak was measured, as well as the angular gap between the (100), and the (010),(110) peaks.

### 2.5. Differential Scanning Calorimetry

The thermal analyses were performed with a PerkinElmer (Waltham, MA, USA) DSC4000. Samples were taken from the surface and the core of the plaques, weighted with a precision balance (with a display accuracy of 1 µg), and then placed in some aluminum pans. From one experiment to another the mass of the samples could vary from 2 mg to 5 mg. The samples were heated at a 10 °C/min rate, from 20 °C to 300 °C, in order to reach their complete fusion. Calibrations were performed with indium and zinc. The typical mass of the samples was around 2 mg.

### 2.6. Dynamic Mechanical Analysis

Dynamic mechanical analyses were performed with a tensile set-up on a PerkinElmer (Waltham, MA, USA) DMA 8000. The tests were performed with a $4\times 10^{-4}$ strain. The α relaxation temperature ($T_{\alpha }$) was determined from temperature scans at 1 Hz with a 2 °C/min temperature rate. Some master curves used for the time-temperature superposition principle were built from frequency scans between 0.1 Hz and 10 Hz. To build the master curves, frequency sweeps were performed every 5 °C at 40 stabilized temperatures from the glassy to the rubbery plateau. For each aging condition, the $T_{\alpha }$ temperatures were chosen as the reference temperature for the master curves.

### 2.7. Tensile Testing

**Figure 3:**
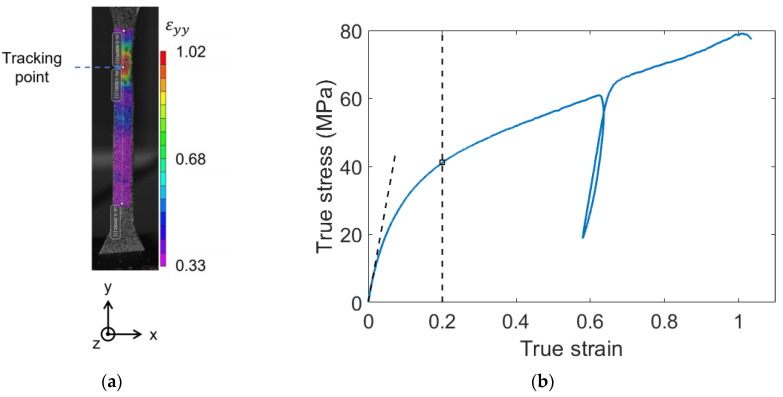
Typical analysis of a tensile test. PA66 taken at the skin of the plaque, aged during 168 h at 120 °C; (**a**) Strain field measured by DIC, and location of the tracking point; (**b**) Measurement of the elasticity modulus and the stress for a 0.2 strain. The strain was measured on the tracking point.

The large strain mechanical behavior was explored by performing some temperature-controlled tensile tests with a 3400 Serie Instron (Norwood, MA, USA) electromechanical device equipped with a 30 kN load cell and an oven. The tests were performed with a $5.0\times 10^{-4}s^{-1}$ strain rate. A digital image correlation (DIC) system was used to track the local displacement and true strain fields (ε) over time. The DIC system was composed of two cameras in stereo-correlation. For each of the tensile specimens, one face was speckled with black and white temperature-resistant paints. The local displacement and strain fields were calculated by analyzing the evolution of the random speckle patterns with the analysis software VIC-3D—Correlated Solutions. A tracking point was then positioned in the maximum strain area. The mechanical behavior was investigated by measuring the strain at the tracking point, i.e., at the maximum of the strain field. A typical strain field, and the location of the tracking point are depicted in Figure 3a where $\varepsilon_{yy}$ is the strain in the tensile direction. The true stress was calculated within the frame of the transverse isotropy assumption, given by Equation (1).

(1) $$\sigma =\frac{F\left(t\right)}{S\left(t\right)}=\frac{F\left(t\right)}{l_{0}\times e_{0}\times exp\left(2\varepsilon_{xx}\right)}$$

where σ is the stress, F(t) is the strength, S(t) is the section of the tensile test specimen, $l_{0}$ and $e_{0}$ are respectively the initial width and the initial thickness of the specimen. $\varepsilon_{xx}$ is the local true strain measured by the tracking point, in the width direction of the specimen. As depicted in Figure 3b, for every tensile test, an elasticity modulus was measured, as well as the true stress at a 0.2 strain. As the stress vs. strain curves do not display a real linear elastic domain, the elastic modulus was calculated by a linear regression in a range of true strain arbitrarily set from 0 to 0.01. A partial unload was introduced between 0.35 and 0.65. On the stress-strain curves, the partial unload leads to the occurrence of a hysteresis loop. Finally, the master curves built by DMA were used to set the loading temperatures and strain rates by referring to the time-temperature superposition principle [31,32,33].

## 3. Results and Discussion

### 3.1. Microstructural Evolution

#### 3.1.1. Average Molar Mass

**Figure 4:**
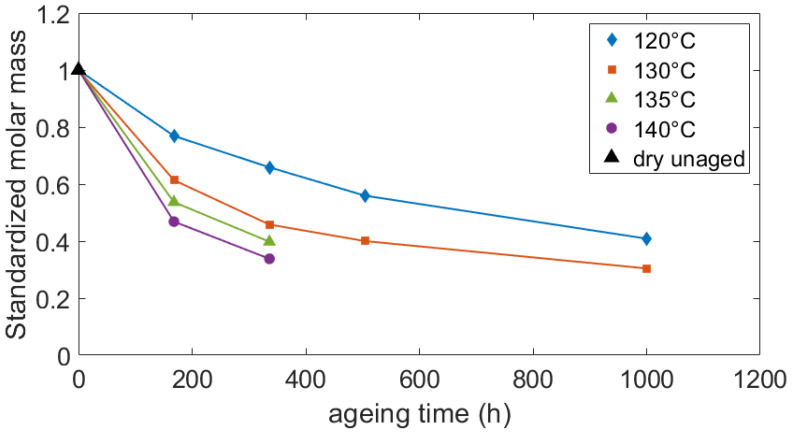
Reduction of the average molar masses due to the hydrolysis and/or oxidation reaction during the ageing processes.

As mentioned before, during the ageing process, the water and ethylene glycol molecules spread into the amorphous phase of PA66 until saturation. In the range of considered temperatures, the hydrolysis reaction occurred (but not glycolysis), resulting in the chain scission of the polymer. Its effects were measured by the decrease of the average molar mass with the aging time (Figure 4). In addition to the hydrolysis reaction, the chain scission can also be a consequence of the oxidation reaction. As already mentioned, the identification of the chemical reactions and the study of their kinetic was not the objective of this paper. The standardized average molar masses measured by the company DOMO Chemicals are depicted in Figure 4. Whatever the chemical reaction (hydrolysis and/or oxidation), the chain scission is faster as the ageing temperature increases. These results are consistent with the literature [2,16]. At the maximum, the molar mass decreased by 65%. This decrease was reached after a 336 h long ageing at 140 °C. For all the ageing conditions, the polydispersity index remained close to 2. It reveals a homogeneous random chain scission phenomenon and the absence of a crosslinking effect due to oxidation. Indeed, when the chains are randomly cut, the number of average molar mass and the weight of average molar mass decrease with the same proportion.

All the results displayed in Figure 4 arise from samples taken in the whole thickness of the plaques, containing core and skin areas. However, after 336 h of aging at 120 °C, specific measurements were also performed in the core and skin areas separately. The resulting standardized molar masses are 0.660 at the core and 0.657 at the skin. The gap between these two measurements is under 0.5% indicating that after aging, the molar mass is homogeneous in the thickness of the plaques. Although oxidation is shown to be a diffusion-limited reaction [38], the present result means that for 1 mm thick areas (core and skin areas), the possible diffusion-limited phenomena are neglectable if the whole bulk of the samples is considered.

#### 3.1.2. Microscopy Observations

**Figure 5:**
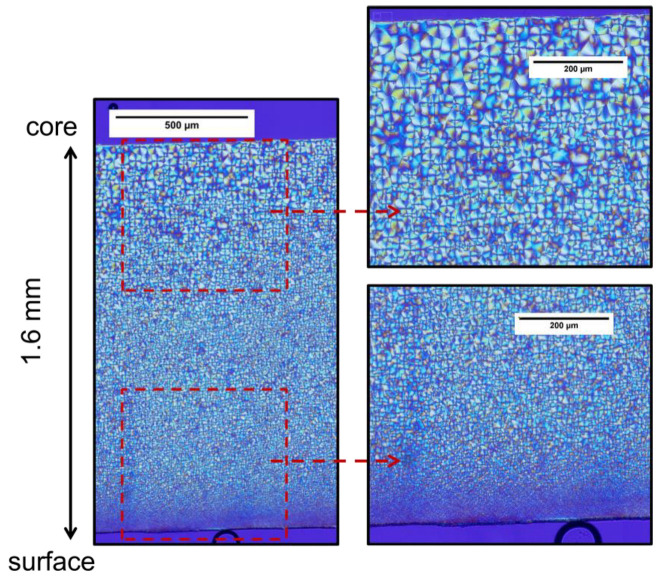
Polarized light microscopy observation of the spherulites for a 140 °C 168 h aged PA66. The picture on the left depicts half of the thickness of the plaque.

Microscopy was used to assess the microstructure at the crystallite scale. A typical polarized light microscopy picture is shown in Figure 5. The spherulites can be easily identified by their Maltese cross pattern. The picture brings out a microstructure gradient: spherulite globally becomes smaller from the core to the surface.

**Figure 6:**
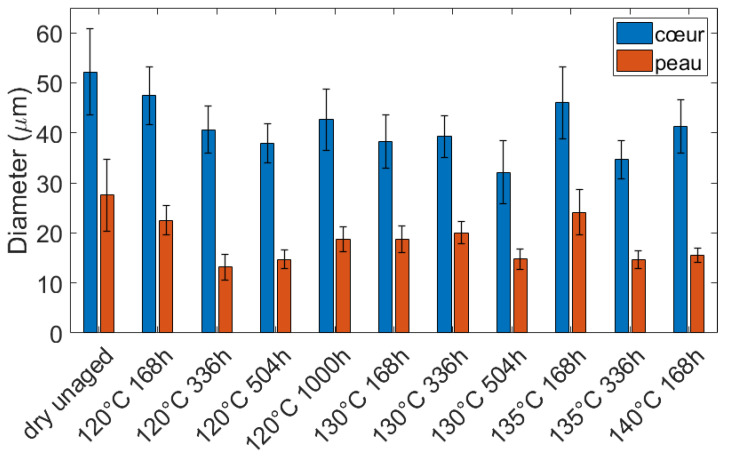
Spherulite diameters were measured from microscopy observations.

To estimate the sizes of spherulites, around 15 spherulites were manually selected near the core and 15 near the surface. Their area was calculated with ImageJ software, and then their diameters for the same area were deduced. This was reproduced for each condition (the initial one and the aged ones). The diameter of the core and the skin spherulites are reported in Figure 6. The differences observed between aged samples must not be interpreted as resulting from aging. They may arise from variations in the measurements. Since these variations are quite small, it ensures that the selected spherulites were representative of the whole spherulite population. Hence, an average diameter of over 165 test points was estimated at the core and at the skin of the plaques (15 measurements per aging conditions). The average diameter is around 40 μm at the core, and around 20 μm at the skin. This gradient clearly arises from the injection molding process. Indeed, the crystallization of polymers and especially the nucleation and the spherulites growth, is well-known to depend on the crystallization temperature and the flowing of the polymers [39,40]. By construction, the injection molding process generates a gradient of thermomechanical history. The microstructure may hence be different depending on its location in the mold.

#### 3.1.3. DSC Results

**Figure 7:**
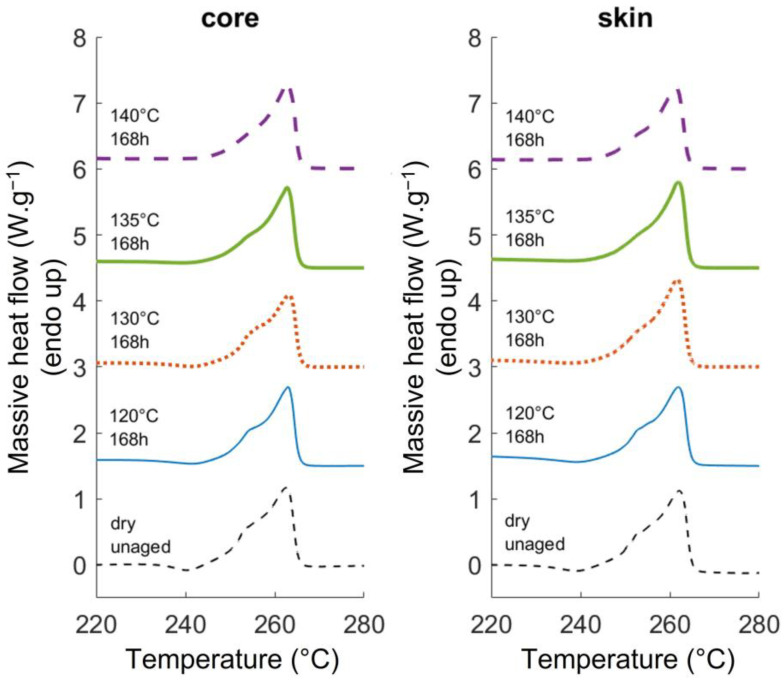
Typical fusion peaks of unaged and 168 h aged PA66.

Typical PA66 fusion peaks are displayed in Figure 7. The melting temperature (the temperature at the maximum of the peak) ranges between 261 °C and 263.5 °C. Near 240 °C, a small exotherm can be observed but its origin is not well determined. It is sometimes attributed to a crystalline reorganization from unstable crystals to more stable ones [26,41]. This exotherm progressively disappears when the aging temperature increases. It could be interpreted as a reorganization of the crystalline phase occurring during aging. In that case, higher temperatures should favor a greater reorganization. Still, in Figure 7, a shoulder can be observed on the peaks around 255 °C. This well-known asymmetry is sometimes attributed to the fusion of smaller and less perfect crystalline entities, or to the transformation of a mesophase into a crystalline phase [42,43,44]. Nevertheless, whatever the underlying phenomenon, the evolution of this shoulder after ageing, highlights that a microstructural reorganization occurred during the ageing process. More specifically, it seems that the shoulder is less pronounced when increasing the aging temperature. It may indicate that the microstructural reorganization is sensitive to the ageing temperature.

From a more quantitative standpoint, the microstructure was assessed at the lamellae scale by considering the lamellae thickness. It was calculated from the DSC thermograms with the Gibbs-Thomson equation (Equation (2)).

(2) $$T_{f}=T_{f}^{0}\left(1-\frac{2\sigma_{e}}{\Delta H_{f}^{0}\cdot \rho_{c}\cdot L_{c}}\right)$$

where $\sigma_{e}$ is the basal surface energy per unit area, $\rho_{c}$ is the density of the crystal, $\Delta H_{f}^{0}$ is the melting enthalpy of a perfect crystal and $T_{f}^{0}$ is the melting temperature of an infinite crystal. For calculation, the following values were used: $\sigma_{e}=0.0296\;J\cdot m^{-2}$, $T_{f}^{0}=270\;{}^{\circ}C$, $\rho_{c}=1240\;\mathrm{kg}\cdot m^{-3}$ and $\Delta H_{f}^{0}=196\;J\cdot g^{-1}$ [27].

**Figure 8:**
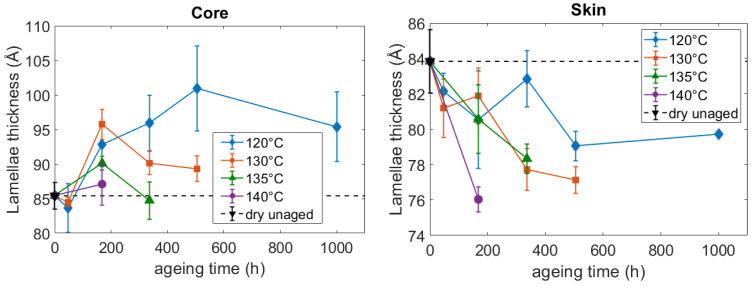
Influence of the aging conditions on the lamellae thickness at the core and the surface of the PA66 plaques.

The evolution of the lamellae thickness is gathered in Figure 8. If one focuses on the average values, the lamellae are initially slightly thicker at the core than at the skin, but the difference is contained in the error bars. Although the initial thickness is similar in the core and skin areas, Figure 8 displays an increase in the thickness at the core and a decrease in the skin. The basic phenomena underlying the evolution of the thickness are difficult to identify, nevertheless, the pre-melting or the refolding of the fold surfaces could be mentioned as well as the melting of the thinner unperfect lamellae [45,46]. The results suggest that depending on the initial microstructure, for the same thermo-hydro-glycol ageing, some phenomena may be preponderant compared to other ones, resulting in opposite evolutions of the lamellae thickness. It was not shown yet in the literature.

#### 3.1.4. WAXS Results

**Figure 9:**
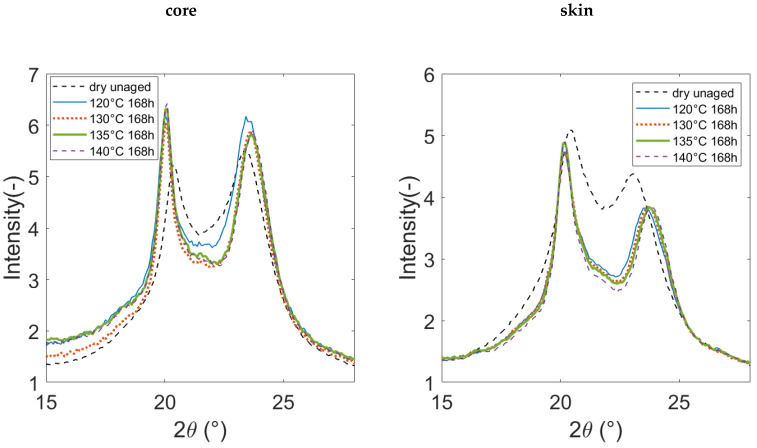
Typical diffractograms of unaged and aged PA66 during 168 h.

Typical diffractograms of unaged and 168 h aged PA66 are gathered in Figure 9. Only a restricted angular window is displayed so that the attention is focused on the two main peaks of the diffractograms. For the unaged polymer, the first main peaks near 20° are higher and wider at the skin than at the core. It may reveal a smaller crystallite’s apparent size. In the core and skin areas, the first main peak gets sharper after aging. Its angular position is also shifted to smaller angles whereas the angular position of the second main peak (near 23°) is shifted to higher angles. These observations are the consequences of microstructural changes occurring during ageing, which will be discussed later.

From the diffractograms of the aged and unaged PA66, the microstructure was again assessed at the lamellae scale bay calculating the crystallite’s apparent size (CAS). It is calculated from the mid-height width of the diffraction peaks by means of the Scherrer equation (Equation (3)), assuming that, θ is the angular position of a crystal peak and Δ2θ is its mid-height width (Figure 2). It corresponds to the dimension of the diffracting crystal entities in the direction normal to the diffracting plans associated to θ.

(3) $$\mathrm{CAS}=\frac{0.9\;\lambda }{\Delta 2\theta \;\cos\theta }$$

**Figure 10:**
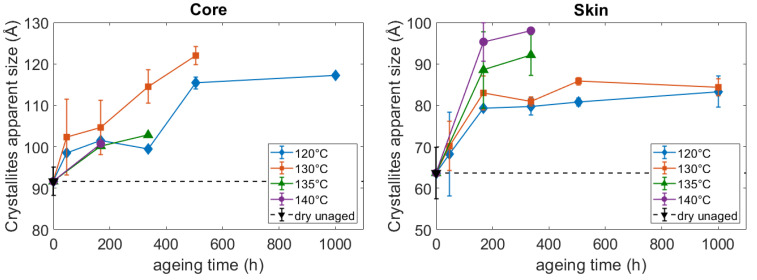
Influence of the ageing conditions on the CAS at the core and at the surface of the PA66 plaques.

The (100) plans and their corresponding peak were chosen to calculate the CAS. Hence the CAS was measured in the H bounding direction, i.e., perpendicular to the chain axis. Figure 10 points out that the diffracting elements are initially and after aging, around 30 Å bigger at the core than at the skin. As for the diameter of the spherulites, these results indicate a microstructural gradient in the thickness of the plaques caused by the injection molding process. After aging, an increase in the CAS can be noticed both at the core and on the skin. It testifies to a reduction of the crystal defects. The resulting lamellae are then composed of bigger diffracting entities. Hence at the lamellae scale, besides modifying the lamellae thickness, the thermo-hydro-glycol ageing also modified the structure of the crystal. More precisely, it resulted in a “perfectioning” of the crystal. The shape of the curves (Figure 10) suggests that the perfectioning reaches an equilibrium over time, and especially after 168 h for the skin. In this specific area, the value of the equilibrium is related to the aging temperature.

**Figure 11:**
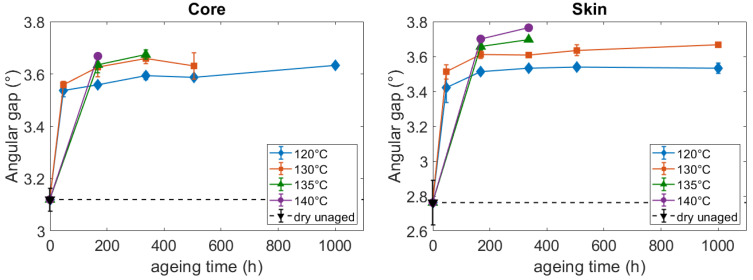
Influence of the aging conditions on the crystal perfection index: the angular gap between the (100) peak and the (010),(001) peak of WAXS diffractograms.

At the crystal cell scale, the microstructure was assessed by measuring a perfection index, referring to the compacity of the crystal. To define it, let us notice that the PA66 crystalline entities are mainly composed of a triclinic α phase [34] which can be refined into a $\alpha_{1}$ phase and a $\alpha_{2}$ phase [35,36]. The density of the $\alpha_{1}$ phase is higher. In other words, the $\alpha_{1}$ phase is more perfect than the $\alpha_{2}$ phase. Because of their different compacity, the diffracting peaks created by these two phases do not appear at the same angular position. Hence Haberkorn et al. [37] identified that the angular gap between the (100) and the (010),(001) peaks (Figure 2) is proportionally related to the amount of $\alpha_{1}$ phase against $\alpha_{2}$ phase. The increase of the angular gap between the (100) and the (010),(001) peaks highlights an increase of the proportion of the $\alpha_{1}$ phase, that is to say, an increase of the packing perfection in the crystalline phase. This angular gap can hence be considered as a crystal perfection index. The measurements brought out that at the dry unaged state, the crystal entities are more perfect at the core than at the skin (Figure 11). Once again, it is a consequence of the injection molding process. After aging, the perfection index increases both at the core and at the skin. The increase is related to the ageing temperature, and it seems to reach an equilibrium after 168 h of ageing. Hence, the results still conclude that thermo-hydro-glycol aging can modify the structure of the crystal of PA66, and more precisely increase its perfection.

The WAXS method was also used to assess the crystallinity of the aged and unaged PA66. A crystallinity index ($\chi_{c}$) was defined as the ratio of the area of the crystalline peaks ($A_{c})$ over the total area of the diffractograms, including the crystalline and amorphous contributions ($A_{a}+A_{c}$) (Equation (4)).

(4) $$\chi_{c}=\frac{A_{c}}{A_{a}+A_{c}}$$

**Figure 12:**
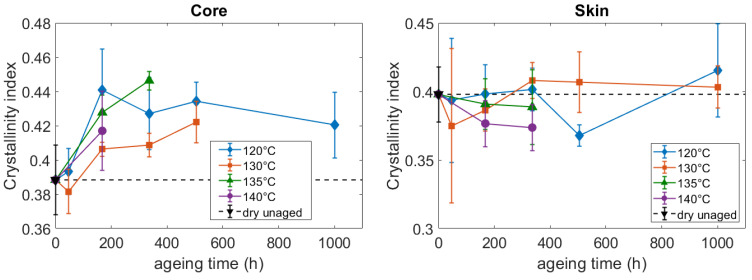
Influence of the ageing conditions on the crystallinity index at the core and the skin of the PA66 plaques.

All the measurements of the crystallinity index are gathered in Figure 12. Before aging, the crystallinity index is very close at the core and to the skin. They both lie around 0.4. Initially, the crystallinity index is a little bit lower at the core, but the difference with the skin is lower than the error bars. After aging, the results bring out some different crystal growth processes occurring at the core and at the skin (Figure 12). At the core, the evolution of the crystallinity index is consistent with the evolution of the lamellae thickness (Figure 8): the growth of the lamellae in their thickness direction results in a higher crystallinity index. At the skin, the results are more difficult to construe. They could be interpreted as the dissolution of the external part of the lamellae into the amorphous phase. If these parts have a too high density of defects, one could assume that they are not detected with the WAXS method. According to this assumption, at the skin, the dissolution of the external part of the lamellae may result in the decrease of the lamellae thickness without changing the crystallinity index. Nevertheless, despite these different tendencies, the values gathered in Figure 12 above all point out that the crystallinity index does not change a lot during aging. Indeed, the average values only change by few percent, and these changes are not significant when compared with the error bars. Hence, as far as the crystalline phase is concerned, the thermo-hydro-glycol ageing does not generate major changes in the amount of crystal, but in the very structure of the crystalline entities.

#### 3.1.5. Microstructural Summary

Finally, microstructural changes caused by thermo-hydro-glycol aging were assessed. A wide range of microstructural parameters was considered, which is a novelty concerning thermo-hydro-glycol ageing. The ageing results in the decrease of the average molar mass due to the hydrolysis and/or oxidation reaction. Concerning the crystalline phase, a core-skin gradient was observed in the PA66 plaques. It is a consequence of the injection molding process. After aging only few changes in the crystallinity index were observed, but the structure of the crystal changed significantly. More precisely significant evolutions of the lamellae thickness were observed, as well as a “perfectioning” of the crystal entities. The main microstructural observations are summarized in Table 1.

### 3.2. Mechanical Consequences

#### 3.2.1. Plasticization

**Figure 13:**
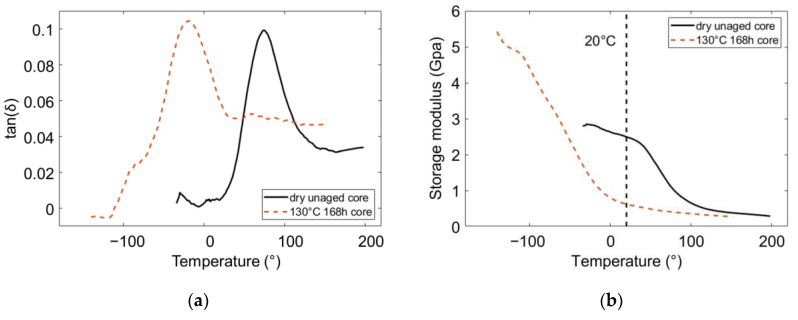
(**a**) Decrease of the damping factor peak to lower temperature after aging; (**b**)Typical storage modulus curves before and after aging.

The intake of small molecules such as water and ethylene glycol, is well-known for resulting in a plasticizing effect. It is characterized by the decrease of the alpha relaxation temperature $T_{\alpha }$. Moreover, some models were developed, to foresee the evolution of $T_{\alpha }$ in a polymer-plasticizer or a polymer-polymer blend [14,15,47,48,49]. Hence after the ageing processes, a decrease of $T_{\alpha }$ was measured by DMA temperature scans. $T_{\alpha }$ was identified as the temperature of the damping factor peak. This phenomenon is illustrated in Figure 13a, where after a specific ageing condition, the damping factor peak is shifted to a lower temperature. All the measured $T_{\alpha }$ are reported in Table 2. Hence, after ageing $T_{\alpha }$ decreases from around 70 °C for a dry unaged PA66, to −20 °C for the aged ones. The exact amount of water and ethylene glycol in the aged samples was not calculated. Karls-Fisher analysis could be set up to do so. Nevertheless, as all the aged PA66 got approximately a 90 °C decrease of $T_{\alpha }$, it suggests that they all contain the same amount of water and the same amount of ethylene glycol.

In addition to the plasticization effect, the glassy plateau of the aged PA66 was shifted to lower temperatures and a much higher modulus (Figure 13b). This phenomenon could be interpreted as an antiplasticization effect [10,50]. This effect was so strong that the glassy plateau was not observed for the aged PA66 in this study, even at −150 °C. No lower temperatures were considered, as −150 °C is already close to the minimum of the operating range of the machine.

The two storage modulus curves displayed in Figure 13b point out that if an aged and a dry unaged PA66 are loaded at the same temperature and the same strain rate, they may not be loaded in the same state. For example, at 20 °C and 1 Hz, the dry unaged PA66 is in a glassy state, whereas the aged one is in a rubbery-like state (see the vertical dashed line). In order to load all the polymers in the same state, an additional drying of the aged samples could have been considered. However, this method has two major drawbacks:

- The additional drying could again modify significatively the microstructure of the aged samples;
- It would avoid eventual mechanical consequences due to the presence of water and ethylene glycol other than plasticization.

**Figure 14:**
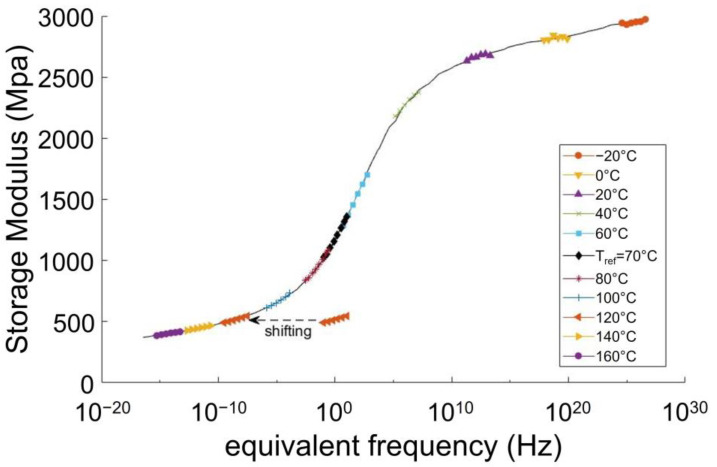
Illustration of some frequency scans used for building a master curve. Example of a dry unaged PA66. For better readability, only a few temperatures are displayed.

Hence, in order to load all the PA66 in the same state, without resorting to additional drying, an approach referring to the time-temperature superposition principle was set up. For every aged PA66 as for the dry unaged one, a master curve was built at a reference temperature, for the core material and the skin materials. The master curves were built by performing frequency scans at several fixed temperatures. For every temperature, a horizontal shift factor was defined to fit with the curve obtained at the reference temperature (Figure 14).

For all the considered PA66, the reference temperature was defined as $T_{\alpha }$: 70 °C for the dry unaged PA66, and −20 °C for the aged ones. Using the master curves, some loading conditions were identified in order to load both the aged and dry unaged PA66 in their rubbery-like state [31,32,33].

**Figure 15:**
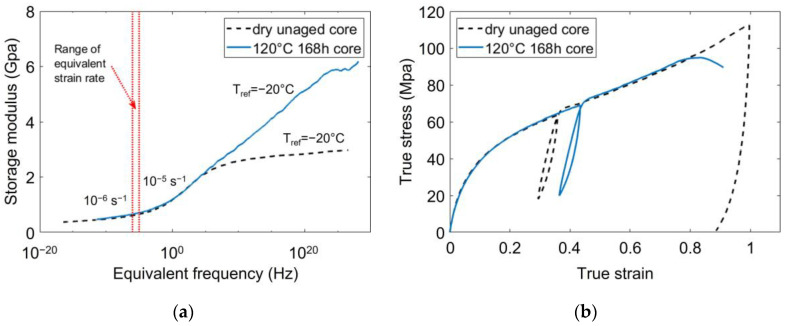
Superposition of two typical master curves. The dry unaged PA66 and the aged one are both loaded on their rubbery plateau. The corresponding strain-stress curves are plotted on the right.

Hence, the dry unaged PA66 were loaded at 80 °C and $5\cdot 10^{-4}s^{-1}$, and the aged PA66 were loaded at −5 °C and $5\cdot 10^{-4}\;s^{-1}$. Depending on the master curves, the equivalent strain rate at $T_{ref}=T_{\alpha }$ lied between $10^{-6}\;s^{-1}$ and $10^{-5}\;s^{-1}$. Figure 15a depicts the master curves of a dry unaged PA66 and a PA66 aged during 168 h at 120 °C. They were both taken at the core of the plaques. The range of the targeted equivalent strain rate is indicated on the master curves by the two vertical dotted lines. This range only represents a restricted portion of the rubbery-like plateau, meaning that the loading conditions are very similar.

The corresponding strain-stress curves are depicted in Figure 15b. They were obtained with a dry unaged PA66 and a 120 °C/168 h aged PA66 taken at the core, loaded with the above-mentioned conditions. As the resulting mechanical behaviors are very similar, the following statements can be confirmed:

- Referring to the time-temperature superposition principle is a relevant approach to foresee the mechanical behavior of PA66 and to set the loading conditions;
- Thanks to this approach all the PA66 considered in this survey were loaded in a rubbery-like state without resorting to an additional drying of the aged specimens.

#### 3.2.2. Impact of the Molar Mass Reduction

**Figure 16:**
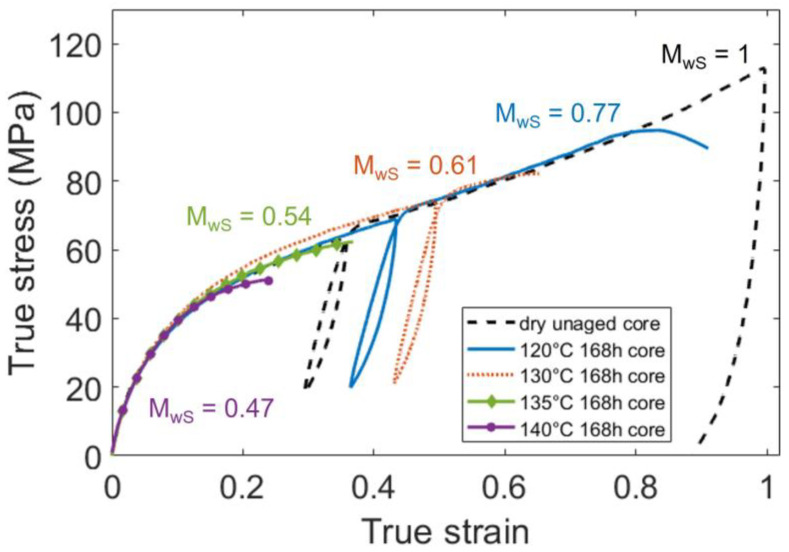
Strain-stress curves of PA66 aged during 168 h, and a dry unaged PA66. Specimens taken at the core of the plaques.

**Figure 17:**
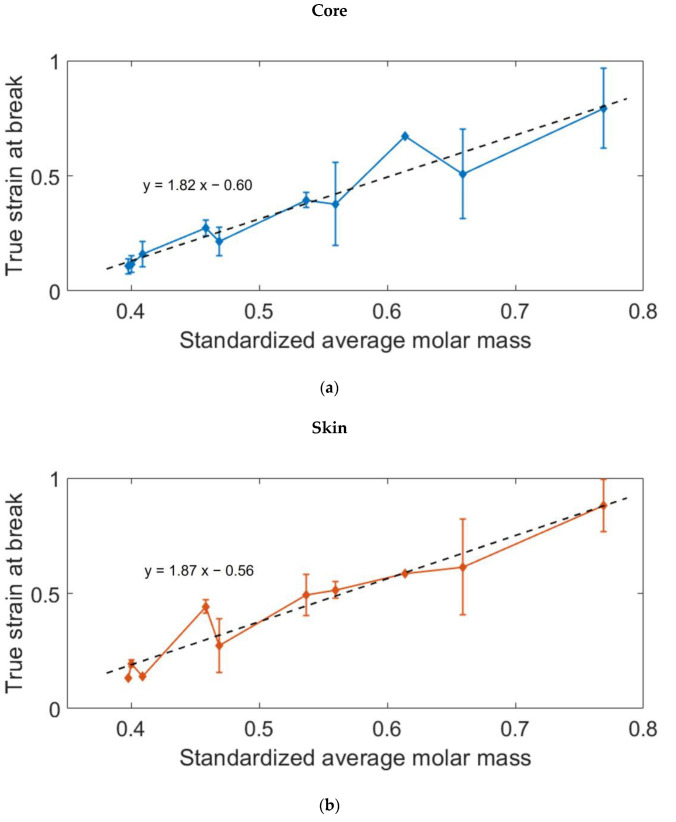
Evolution of the true strain at the break with the decreasing molar mass. Measurements and tendencies: (**a**) at the core; (**b**) at the skin.

The large strain mechanical behavior of the aged and unaged PA66 was assessed with two or three tensile tests for each condition. Figure 16 depicts the stress-strain curves of PA66 aged during 168 h and a dry unaged PA66, taken at the core of the plaques. As mentioned above, all the PA66 display almost the same mechanical behavior, corresponding to the rubbery-like plateau. For the aged samples, a breaking occurred before the final unload. A strain at break was then measured, and the results showed that the embrittling of the polymer was related to the reduction of the molar mass. Figure 17 gathers the strain at break measured on every aged specimen. They are plotted as a function of the corresponding standardized molar mass. It clearly brings out that the true strain at break is driven by the average molar mass. More precisely, the molar mass reduction occurring during the ageing process results in the embrittling of the material, both at the core and at the surface. Moreover, this relationship appears to be linear, and almost the same slope is obtained at the core and at the skin. It means that compared to the average molar mass, the crystalline differences between the core and the skin have a neglectable influence on the ductility of the polymer.

These results are consistent with those from the literature [2,20,51], although most of the studies dealing with the thermo-hydro-glycol ageing of PA66 consider engineering strain and not the local true strain.

#### 3.2.3. Impact of the Crystal on the Stiffness

**Figure 18:**
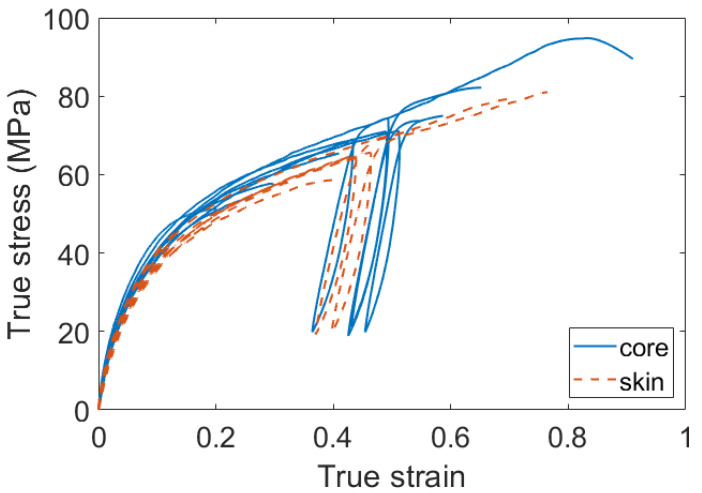
Illustration of the gradient of stiffness between the core and the skin material. Two curves are displayed for every aging condition: one for the core and one for the skin.

Finally, the impact of the crystal properties on the large strain mechanical behavior was also investigated. For every aging or dry unaged condition, it was observed that the core material was always stiffer than the skin material. This observation is illustrated in Figure 18. In this figure, two stress-strain curves are displayed for every ageing condition: one for the core material and one for the skin material. It can be observed that globally, the core material is stiffer than the skin material.

At the beginning of the tensile tests, the gradient of stiffness can be observed by comparing the elastic modulus. They are all reported in Table 3. Except for the PA66 aged during 336 h at 130 °C and 135 °C, the elastic modulus is always higher at the core than at the skin. However, for the above-mentioned condition, the difference between the core and the skin is not higher than some tens of MPa, i.e., almost the accuracy of the measurement. These two unexpected observations can hence be attributed to the variability of the tests.

**Figure 19:**
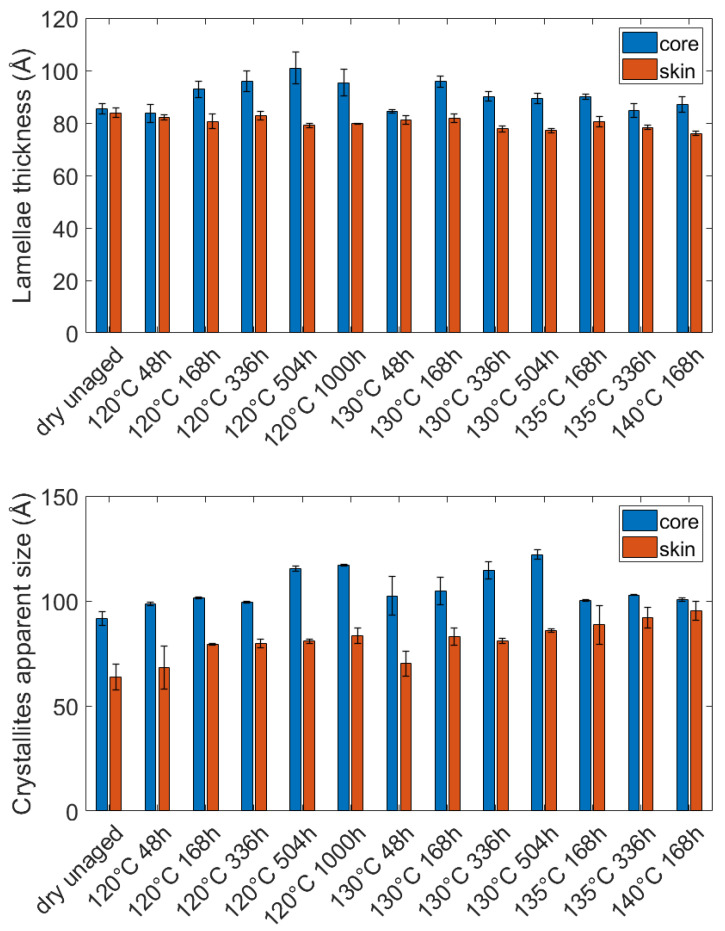
Comparison of the dimension of the crystalline entities at the core and at the surface: Lamellae thickness and crystallites apparent size.

Moreover, this gradient of stiffness comes along with a gradient of microstructure. As the crystallinity index is almost the same at the core and at the skin (Figure 12), the gradient of stiffness may be caused by the very structure of the crystal. As displayed in Figure 6 and Figure 19, the dimensions of the crystalline entities are always bigger at the core than at the skin, especially the spherulites diameter, the lamellae thickness, and the crystallite’s apparent size. Hence, it can be stated core/skin gradient of microstructure generated by the thermo-hydro-glycol ageing results in a gradient of stiffness with a core material stiffer than the skin material.

**Figure 20:**
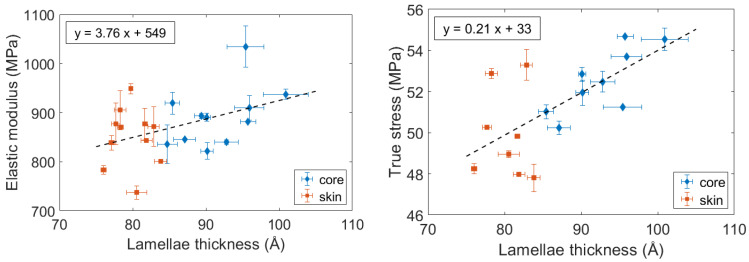
Influence of the lamellae thickness on the PA66 stiffness: Elastic modulus and true stress at a 0.2 strain.

The influence of the lamellae thickness was then more precisely investigated, and not limited to a comparison between the core and the skin. For every ageing condition, the elastic modulus, and the measured stress at 0.2 were plotted as a function of the corresponding lamellae thickness (Figure 20). It appears that an increase in the lamellae thickness results in an increase in the stiffness. A linear tendency can be identified, both for the elastic modulus and the stress at 0.2. The relationship between the lamellae thickness and the stiffness could be explained by referring to the constraint level of the interlamellar amorphous phase. One could assume that as the lamellae are thicker, the amorphous phase is more constrained, leading to a stiffer mechanical behavior.

#### 3.2.4. Mechanical Summary

The mechanical behavior of the aged and unaged PA66 was assessed by tensile testing. It seems to depend above all on the state of the polymer, which depends itself on the gap with $T_{\alpha }$. To avoid the plasticizing effect caused by the water and ethylene glycol intake, an approach referring to the time-temperature superposition principle was set up. All the PA66 were then loaded in a rubbery-like state. For the same state it was pointed out that the brittleness of the polymer is primarily driven by its average molar mass. Still, for a same state, changes in the crystalline structure have a non-negligible influence on the stiffness of the polymer, and especially the lamellae thickness. This influence is the reason behind the observation of a core/skin gradient of stiffness. The main microstructure-properties relationships are summarized in Table 4.

## 4. Conclusions

A wide range of experimental methods was used to assess the evolution of the microstructure and the mechanical behavior of an injection molded PA66 over thermo-hydro-glycol aging. The objective was to give a complete view of the microstructural evolutions occurring during aging, and to analyze their mechanical consequences. The microstructure-properties relationships were hence investigated. To take into account that the injection molding process does not generate a homogeneous microstructure, a core/skin approach was set up.

Concerning the microstructure, a decrease in the average molar mass was measured. It is related to the occurrence of the hydrolysis and/or oxidation reaction. Moreover, the measures of the polydispersity index assert the random chain scission of the polymer. The objective of this paper was not to determine the exact nature or kinetics of the chemical reaction, but rather to focus on its microstructural consequences.

In addition to the molar mass measurements, the evolution of the crystalline phase was investigated. The amount of crystal was discussed, as well as the structure of the crystal itself, which is a novelty concerning thermo-hydro-glycol ageing. To do so the structure was assessed from the spherulite scale to the crystal cell scale. Before and after ageing, a microstructural gradient was observed in the thickness of the specimens as a consequence of the injection molding process. The thermo-hydro-glycol aging caused very few changes in the crystallinity index whereas the structure of the crystal changed significatively. At the core, the lamellae thickness increased whereas it decreased at the skin. It means that in addition to the core/skin gradient, the opposite phenomena can also occur in the thickness of the samples during the thermo-hydro-glycol aging, and not only at the extreme surface as it is for example in the case of diffusion-limited reactions [38]. Finally, both at the core and at the skin, the thermo-hydro-glycol aging resulted in the “perfectioning” of the crystal attested by an increase of the crystallite apparent size and the crystal perfection index. The increase of these two parameters is construed by respectively by a decrease in the density of defects in the crystal, and an increase in its compacity. Even though it was not the objective of this paper, it can be said, that it was difficult to identify the specific role of water, ethylene glycol, and the heat treatment in the changes in the crystalline phase. To do so, specific aging in only water, ethylene glycol, or in a controlled atmosphere could be considered.

The mechanical behavior of PA66 was investigated by tensile testing. The first consequence of aging was the plasticization of the polymer caused by the water and ethylene glycol intake. Around a 90 °C decrease of $T_{\alpha }$ was measured for all the aged PA66. To load plasticized polymers in the same state as that of the dry unaged ones, the loading conditions were chosen by referring to the time-temperature superposition principle. All the aged and unaged PA66 were then loaded in a rubbery-like state. This method was found relevant to do so without resorting to additional drying.

The results showed that the mechanical behavior of the aged and unaged PA66 is above all related to the state of the polymer. Indeed, for the same rubbery-like state, all the tested polymers had similar mechanical behavior. Still, for a same state, the ductility of PA66 is driven by its average molar mass, hence its reduction due to hydrolysis/oxidation led to the embrittlement of PA66. Finally, the changes in the structure of the crystal occurring during the aging were found to have an impact on the stiffness of the polymer, especially for the same rubbery-like state; the stiffness is related to the lamellae thickness. This influence of the crystalline structure is the cause of a gradient of stiffness observed in the thickness of the samples.

## Figures and Tables

**Table 1:** Summary of the microstructural changes occurring in an injection molded PA66 during thermo-hydro-glycol ageing.

Measurement Scale	Main Results
Molar mass	Reduction of the average molar mass due to the hydrolysis reaction
	No core/skin gradient.
Spherulites diameter	Core/skin gradient.
Crystallites’ apparent size	Increase of the crystallites’ apparent size at the core and at the skin.
	Core/skin gradient.
Lamellae thickness	Thickening of the lamellae at the core of the plaques.
	Reduction of the thickness of the skin.
Crystal perfection index	Increase of the perfection at the core and at the skin.
	Core/skin gradient before aging.
Crystallinity index	Few changes in the crystallinity index.
	Small increase at the core of the plaques.

**Table 2:** $T_{\alpha }$ for the dry unaged and the aged PA66.

Aging Condition	$T_{\alpha }\;\mathrm{in}\;\mathrm{the}\;\mathrm{Core}$	$T_{\alpha }\;\mathrm{in}\;\mathrm{the}\;\mathrm{Skin}$
Dry unaged	70 °C	70 °C
120 °C 168 h	−19 °C	−18 °C
120 °C 336 h	−19 °C	−19 °C
120 °C 504 h	−20 °C	−19 °C
120 °C 1000 h	−20 °C	−17 °C
130 °C 168 h	−19 °C	−20 °C
130 °C 336 h	−21 °C	−20 °C
130 °C 504 h		−19 °C
135 °C 168 h	−22 °C	−20 °C
135 °C 336 h	−19 °C	−20 °C
140 °C 168 h	−20 °C	−18 °C

**Table 3:** Elastic modulus of the aged PA66 end the dry unaged one. Measurements on specimens taken at the core and at the skin of the plaques.

Aging Condition	Core	Skin
Dry unaged	920 MPa	800 MPa
120 °C 168 h	840 MPa	740 MPa
120 °C 336 h	910 MPa	870 MPa
120 °C 504 h	940 MPa	910 MPa
120 °C 1000 h	1030 MPa	950 MPa
130 °C 168 h	880 MPa	840 MPa
130 °C 336 h	820 MPa	880 MPa
130 °C 504 h	890 MPa	840 MPa
135 °C 168 h	890 MPa	880 MPa
135 °C 336 h	840 MPa	870 MPa
140 °C 168 h	850 MPa	780 MPa

**Table 4:** Microstructure-properties relationships observed in an aged injection molded PA66.

Microstructural Observations	Mechanical Consequences
Water and ethylene glycol intake	$\mathrm{Plasticization}\;\mathrm{of}\;\mathrm{PA}66:\;\mathrm{decrease}\;\mathrm{of}\;T_{\alpha }$
Decrease of the average molar mass	Embrittlement of the polymer
Core-skin gradient: bigger crystal entities at the core	Core stiffer than the skin
Evolution of the lamellae thickness	Evolution of the stiffness: thicker lamellae result in a stiffer polymer.
